# Endothelial Cell-Derived Extracellular Vesicles Target TLR4 via miRNA-326-3p to Regulate Skin Fibroblasts Senescence

**DOI:** 10.1155/2022/3371982

**Published:** 2022-05-18

**Authors:** Xinni Yang, Jiyong Tan, Jiqing Shen, Xin Zhang, Gaoxiang Huang, Xiaoxue Su, Jing Li

**Affiliations:** ^1^School of Basic Medicine, Guangxi Medical University, Nanning, Guangxi 530021, China; ^2^Key Laboratory of Longevity and Aging-Related Diseases of Chinese Ministry of Education, Nanning, Guangxi 530021, China; ^3^The First Affiliated Hospital of Jinzhou Medical University, Jinzhou, Liaoning 121000, China; ^4^Guangxi Colleges and Universities Key Laboratory of Biological Molecular Medicine Research, Guangxi Medical University, Nanning 530021, China; ^5^Guangxi Colleges and Universities Key Laboratory of Basic Medicine Research, Guangxi Medical University, Nanning 530021, China

## Abstract

**Backgrounds:**

Skin aging could be regulated by the aberrant expression of microRNAs. In this manuscript, we explain that endothelial cell-derived extracellular vesicles could act as supporters to deliver exogenous miR-326-3p to accelerate skin fibroblasts senescence.

**Methods:**

*β*-galactosidase senescence staining assay, Hoechst 33258 apoptosis staining assay, and Ki67 staining assay were used to evaluate the biological function of mouse skin fibroblasts. Real-time PCR was applied to assay miRNAs and mRNAs expressions. Western blot was used to detect TLR4 protein expression. The target gene of miRNA were identified using a double luciferase reporter assay. miR-326-3p mimic/inhibitor and siRNA-TLR4 can demonstrate a nonnegligible link between miR-326-3p-TLR4 and skin aging.

**Results:**

In coculture experiment, senescence endothelial cells could promote the skin fibroblasts senescence and apoptosis via extracellular vesicles pathway. In contrast, miR-326-3p mimics accelerated senescence and apoptosis of skin fibroblasts, while miR-326-3p inhibitor could dramatically delay skin fibroblasts senescence and apoptosis. TLR4 was proved to be a miR-326-3p directly target gene via double luciferase assay. After skin fibroblasts transfected with siRNA-TLR4, cellular senescence and apoptosis were significantly increased. Furthermore, the skin tissues of aging mice were shown with overexpression of miR-326-3p and decrease of TLR4 gene and protein expression levels.

**Conclusions:**

Endothelial cell-derived extracellular vesicles delivery of miR-326-3p was found to have a function in skin fibroblasts via target TLR4. Therefore, endothelial cell-derived extracellular vesicles in antiaging therapies might be a new treatment way for delaying skin aging.

## 1. Introduction

Healthy skin segregates the body from the outer environment, a significant barrier protecting the body from water loss. Fossilized skin shows visible signs such as wrinkling, loss of elasticity, and laxity [1]. The barrier function of older people's skin is greatly reduced, their skin gradually becomes dry and cracked, and they are at increased risk of skin diseases [2]. After skin aging, there is a high chance of skin malignancies [3]. Currently, the mechanism of cell-to-cell communication for skin microenvironment during aging process is still unclear. An extensive understanding about the skin aging process will provide valuable evidence and insights for developing new skin protection production that may reduce the aging process and prevent risk factors of skin aging [4, 5].

Extracellular vesicles, including of microRNA (miRNA), long noncoding RNA, and circular RNA, are considered as mediators for intercellular communication [6–9]. Extracellular vesicles play an important role in various physiology and pathology processes, such as cancer, cardiovascular diseases, neuropsychiatric disorders, and metabolic disease [10]. miRNAs are involved in many processes of cell biology, and they can be involved in all processes one can think of [11–15]. For example, miR-146a could inhibit UVA-induced photoaging by targeting Smad4 [16]. miR-217 targeted DNA methyltransferase 1 to regulate skin fibroblast cells senescence process [17].

In our previous study, we found that miR-302b-3p could speed up skin aging procedure through targeting JNK2 gene [18]. Additionally, we found that UV radiation can hurt endothelial cells and accelerate skin photoaging, while protection of skin endothelial cells could delay skin photoaging during UV radiation. But its mechanism is not clear. Now we demonstrated that endothelial cell-derived extracellular vesicles had the quality of cellular vehicles to deliver exogenous miR-326-3p and accelerate skin fibroblasts senescence via targeting TLR4.

## 2. Methods

### 2.1. Animals

The experimental procedures have been approved by the Institutional Animal Ethics Guidelines for the Study of Animal Care and Use established by Guangxi Medical University. Particularly pathogen-free male C57BL/6 mice (Laboratory Animal Centre, Guangxi Medical University), 6-week-old, were housed in acrylic cages in an animal room. After 7-day initial acclimatization period, mice were randomized into two groups and given a daily subcutaneous injection of PBS or 200 mg/kg dosages of D-galactose for a period of 12 weeks. After anesthesia with isoflurane, all animals started cervical dislocation and then sacrificed.

### 2.2. Cell Culture

Primary fibroblasts from the tergal derma of 5-day-old newborn mice were used as a cellular model in this study. All the procedures were performed according to the isolation protocol of skin fibroblasts in our laboratory [18]. Mouse lung microvascular endothelial cell line was got from Shanghai Aolu company (China).

### 2.3. Senescence *β*-Galactosidase Staining Assay

After being fixed in *β*-galactosidase fixative for 15 minutes, the cells of SA-*β*-gal positive presented with a green color. Then, calculate the percentage of staining cells.

### 2.4. Real-Time PCR Assay

Cells were collected and lysed using TRIzol reagent. The expression level of miR-326-3p was detected by reverse transcription with the Mir‐X miRNA First Strand Synthesis Kit. For mRNA analysis, reverse transcription into cDNA was conducted with the RevertAid 1st Strand cDNA Synthesis Kit. The miR-326-3p and TLR4 mRNA expression levels were standardized to snoU6 and GAPDH expression. [19] The primer lists used in this manuscript were shown as follows: mmu-miR-326-3p upstream primers: CCUCUGGGCCCUUCCUCCAGU; TLR4 upstream primer: 5′-ACAAGGCATGGCATGGCTTACAC-3′; downstream primer: 5′-TGTCTCCACAGCCACCAGATTCTC-3′; GAPDH upstream primer: 5′-GGTTGTCTCCTGCGACTTCA-3′; downstream primer: 5′-TGGTCCAGGGTTTCTTACTCC-3′.

### 2.5. Hoechst 33258 Staining

Use Hoechst 33258 staining to detect apoptosis. After 30 minutes, the cells were added with Hoechst 33258 fluorescent dye for 10 min. Apoptotic nuclei were observed to be blue under a fluorescent microscope. Images were familiar with a fluorescence microscope (Leica, Germany) at an excitation wavelength of 340 nm.

### 2.6. Extracellular Vesicles Isolation and Identification

Extracellular vesicles are kept away from the supernatants of endothelial cells by a one-step polymer precipitation procedure using ExoQuick Precipitation Solution, according to the maker's guidance, with incubation at 4°C for 0.5 h and pelleting by centrifugation at 13000 rpm for 2 minutes [20, 21]. 5 *μ*L of purified extracellular vesicles was dropped onto sealing films, stained with 1% phosphotungstic acid-staining droplet for 30 s, and drained sealing film. Extracellular vesicles were observed with a Philips CM120 electron microscope.

### 2.7. Western Blotting

Then the proteins were divided into a 10% SDS-PAGE gel, moved to PVDF membranes, and hatched with primary antibodies TLR4, *β*-actin (1 : 10 000 dilution) at 4°C overnight. Next, the membranes were hatched with peroxide-conjugated antibodies for 2 h. The signal was quantified with Quantity One software. Each experiment must be done more than 3 times.

### 2.8. Dual-Luciferase Reporter Gene Assay

NC-mimic/miR-326-3p mimic or WT-TLR4/MUT-TLR4 plasmids were transiently co-transected with cells in 6-well plates. After treatment 48 hours, luciferase activity of the cells was detected through Dual-Luciferase Reporter Assay System kit (Promega, Madison, USA).

### 2.9. Hematoxylin and Eosin (H&E)

The samples were put in 10% formaldehyde solution, dehydrated in ethanol with gradient concentration, embedded in paraffin, and cut down into various parts. Then, the parts were degreased and stained with hematoxylin and eosin. Finally, every part was observed under a light microscope (Leica, Germany).

### 2.10. Masson Staining

Masson staining (Solarbio, Beijing, China) was performed to identify the content of collagen fiber in the extracellular matrix according to the protocol of the kits. The sections were finally observed by a microscope (Leica, Germany).

### 2.11. Analysis of Statistics

The SPSS software (version 19.0) and GraphPad Prism 6 Software (San Diego, CA, USA) were utilized to analyze the data (mean ± standard deviation (SD)) in this study. Student's *t*-tests were applied to evaluate the difference between groups, while the comparison among multiple groups was conducted by one-way ANOVA. *p* < 0.05 was considered significant.

## 3. Results

### 3.1. Senescence Endothelial Cells Promoted Skin Fibroblasts Senescence via Extracellular Vesicles Pathway

**Figure 1:**
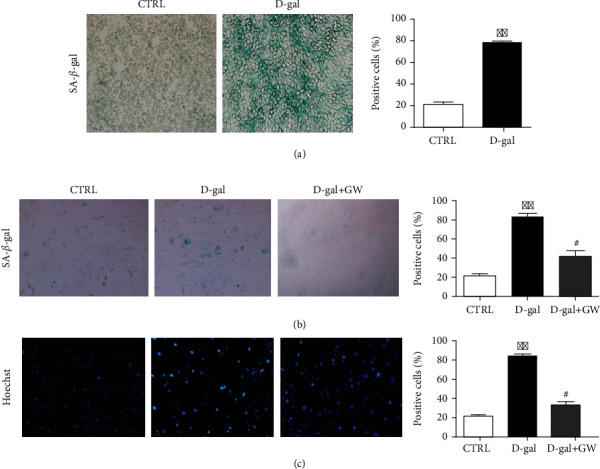
Senescence endothelial cells promoted skin fibroblasts senescence via extracellular vesicles pathway. (a)SA-*β*-gal staining analyze of D-gal-induced senescence endothelial cells. (b) SA-*β*-gal staining assay of skin fibroblasts cocultured with endothelial cells treated by PBS, D-gal, and D-gal + GW4869. (c) Hoechst 33258 staining assay of skin fibroblasts cocultured with endothelial cells treated by PBS, D-gal, and D-gal + GW4869. ∗∗*p* < 0.01 vs. CTRL; #*p* < 0.05 vs. D-gal;. CTRL: control; D-gal: D-galactose; GW: GW4869.

First, a model of senescence endothelial cells was induced with D-galactose (D-gal, 20 g/L) and then stained, from which it can be concluded that D-gal can induce endothelial cell senescence (Figure 1(a)). To estimate the effect of senescence endothelial cells on fibroblasts via extracellular vesicles pathway, the skin fibroblasts were cocultured with control endothelial cells, D-gal-induced senescence endothelial cells, and D-gal-induced senescence endothelial cells with GW4869, respectively. GW4869 is an inhibitor of extracellular vesicles biogenesis/release. We found that the numbers of senescence and apoptosis positive cells in skin fibroblasts were significantly increased after cocultured with senescence endothelial cells. On the contrary, the numbers of senescence and apoptosis-positive cells in skin fibroblast cells cocultured with senescence endothelial cells were significantly decreased after treatment with GW4869. (Figures 1(b) and 1(c)).

### 3.2. miR-326-3p Overexpression in Extracellular Vesicles of Senescence Endothelial Cells

**Figure 2:**
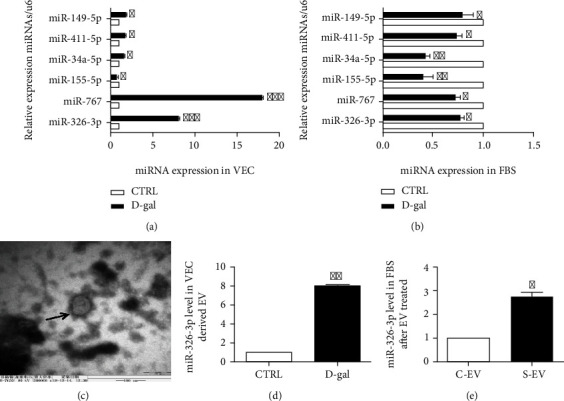
miR-326-3p overexpression in extracellular vesicles of senescence endothelial cells. (a) The expression of miRNAs associated with aging in vascular endothelial cells (VEC). (b) The expression of miRNAs related to senescence in skin fibroblasts (FBS). (c) Electron microscope observation of extracellular vesicles derived from vascular endothelial cells. (d) The miR-326-3p expression in extracellular vesicles (EV) of vascular endothelial cells (VEC). (e) The expression of miR-326-3p in skin fibroblasts after treated with endothelial cell-derived extracellular vesicles. ∗*p* < 0.05 vs. CTRL; ∗∗*p* < 0.01 vs. CTRL. ∗∗∗*p* < 0.001 vs. CTRL. CTRL: control; D-gal: D-galactose; C-EV: control endothelial cell-derived extracellular vesicles; S-EV: senescent endothelial cell-derived extracellular vesicles.

We found that miR-149-5p, miR-411-5p, miR-34a-5p, miR-326-3p, and miR-767 were upregulation and miR-155-5p was low expression in D-gal-induced senescence vascular endothelial cell (VEC) by qRT-PCR (Figure 2(a)), but miR-149-5p, miR-411-5p, miR-34a-5p, miR-155-5p, miR-326-3p, and miR-767 were all low expression in D-gal-induced senescence skin fibroblasts (FBS) (Figure 2(b)). Next, we isolated extracellular vesicles from the supernatants of endothelial cells. Via using electron microscope scanning, a cup-shaped or spherical morphology was observed in extractive of culture supernatants (Figure 2(c)), similar to the extracellular vesicles described previously [22]. We detected miR-326-3p via qRT-PCR and found that the level of miR-326-3p in the extracellular vesicles derived from senescence endothelial cells was significantly increased (Figure 2(d)). To evaluate the effect of senescence endothelial cell-derived extracellular vesicles on skin fibroblasts, the skin fibroblasts were treated with senescence endothelial cell-derived extracellular vesicles or an equal volume of control endothelial cell-derived extracellular vesicles. We found that the grade of miR-326-3p in the skin fibroblasts was significantly enhanced after treatment with senescence endothelial cell-derived extracellular vesicles (Figure 2(e)).

### 3.3. miR-326-3p Regulated Skin Fibroblasts Biological Functions

**Figure 3:**
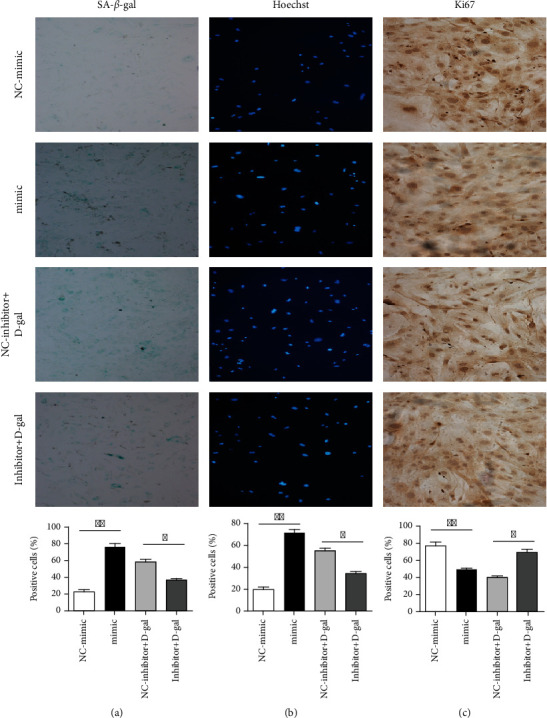
miR-326-3p regulated skin fibroblasts biological functions. (a) SA-*β*-gal staining assay of skin fibroblast cells after treated with miR-326-3p mimic/inhibitor (10×). (b) Apoptosis (Hoechst 33258) staining assay of skin fibroblast cells after treated with miR-326-3p mimic/inhibitor (10×). (c) Ki67 proliferation staining assay of skin fibroblast cells after treated with miR-326-3p mimic/inhibitor (10×). ∗*p* < 0.05 vs. NC, ∗∗*p* < 0.01 vs. NC. NC: negative control.

To further explore the regulation methodology of miR-326-3p during aging, we used miR-326-3p mimic or inhibitor to induce miR-326-3p overexpression or downregulation, respectively. As shown in Figure 3, compared with the control part, the overexpression of miR-326-3p mimic significantly increased the numbers of cellular senescence and apoptosis via *β*-galactosidase staining and Hoechst 33258 staining (Figures 3(a) and 3(b)). On the contrary, the D-gal-induced senescence skin fibroblasts transfected with miR-326-3p inhibitor exhibited a significant reduction in senescence and apoptosis cells. In addition, the expression of miR-326-3p decreased the number of cells proliferation, while the inhibition of mir-326-3p increased the number of cells proliferation (Figure 3(c)).

### 3.4. TLR4 as a Target Gene of miR-326-3p

**Figure 4:**
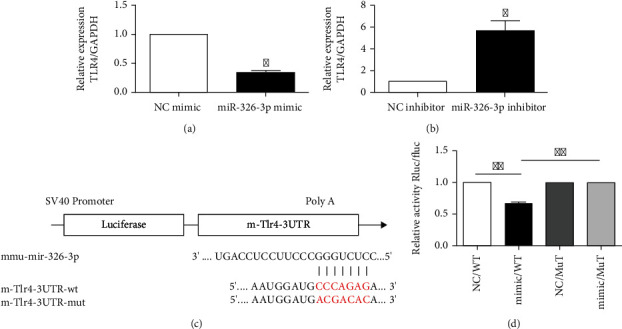
TLR4 as a target gene of miR-326-3p. (a) TLR4 mRNA expression in skin fibroblast cells after transfected with miR-326-3p mimic. (b) TLR4 mRNA expression in skin fibroblast cells after transfected with miR-326-3p inhibitor. (c) Predicted binding sites for miR-326-3p target gene TLR4. (d) Double luciferase assay verified the targeted association between TLR4 and miR-326-3p. ∗*p* < 0.05 vs. NC, ∗∗*p* < 0.01 vs. NC.

TargetScan (http://target-scan.org) predicts TLR4 for miR-326-3p in mice. To help clarify and quantify its inhibitory effect, fibroblasts were also transfected with miR-326-3p mimic or inhibitor. When miR-326-3p mimic was administered, TLR4 mRNA were dramatically decreased, while miR-326-3p inhibitor showed the opposite trend (Figures 4(a) and 4(b)). In double luciferase assay (Figures 4(c) and 4(d)), cotransfection of WT TLR4 3′-UTR with miR-326-3p mimic significantly reduced luciferase activity as compared with the control group. Hence, the administration of miR-326-3p mimic in cells transfected with mutant TLR4 3′-UTR had no effect on luciferase expression.

### 3.5. TLR4 Modulated Skin Fibroblasts Senescence

**Figure 5:**
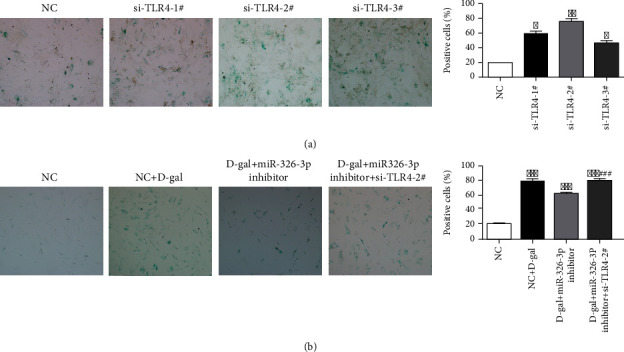
TLR4 modulated skin fibroblasts senescence. (a) SA-*β*-gal staining analysis of skin fibroblasts after transfected with siRNA-TLR4 (10×). (b) SA-*β*-gal assay of skin fibroblasts after cotransfected with siRNA-TLR4 and miR-326-3p inhibitor (10×). ∗*p* < 0.05 vs. NC, ∗∗*p* < 0.01 vs. NC, ∗∗∗*p* < 0.001 vs. NC. ###*p* < 0.001 vs. D-gal+miR-326-3p inhibitor.

To further explore the molecular mechanism of TLR4 in aging process, siRNA-TLR4 was transfected to induce TLR4 downregulation. As shown in Figure 5(a), the down-expression of TLR4 significantly decreased the rates of cellular senescence. Incredibly, miR-326-3p inhibitor could delay D-gal-induced senescence in skin fibroblasts, but miR-326-3p inhibitor transfected with siRNA-TLR4 could not attenuate D-gal-induced senescence in skin fibroblasts (Figure 5(b)), suggesting that miR-326-3p could promote fibroblasts senescence through TLR4 pathway.

### 3.6. miR-326-3p and TLR4 Expression in Aging Skin

**Figure 6:**
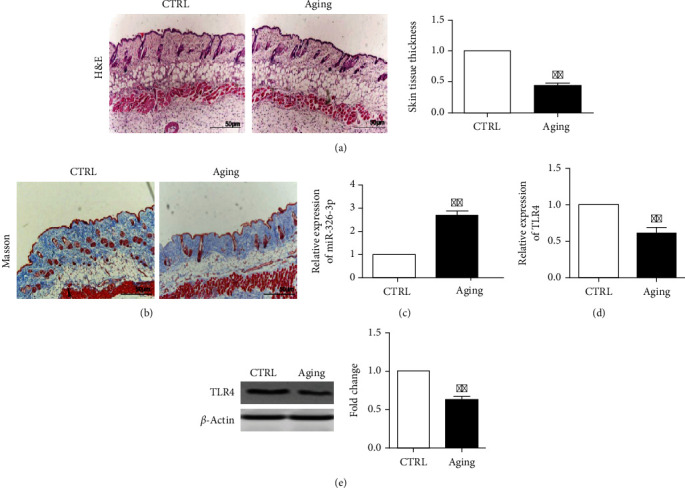
miR-326-3p and TLR4 expression in aging skin tissue. (a) HE staining and dermal thickness analyze of aging mice skin tissue (10×). (b) MASSON staining of aging mice skin tissue (10×). (c) The miR-326-3p expression in aging skin tissue. (d) The TLR4 mRNA expression in aging skin tissue. (e) The expression of TLR4 protein in aging skin tissue. ∗∗*p* < 0.01 vs. CTRL group. CTRL: control.

After D-gal treatment for 3 months, aging mice were showed with decrease in skin thickness and collagen fibers in the extracellular matrix and Masson staining assay (Figures 6(a) and 6(b)). Interestingly, miR-326-3p level is dramatically increased, and TLR4 expression is significantly reduced (Figures 6(c) and 6(d)). The decreased TLR4 protein levels were also shown in aging skin tissue when compared with youth control mice (Figure 6(e)).

## 4. Discussion

Accordingly, many researches have figured out that the potential miRNAs are used in the diagnosis or prognosis of diseases, and the results often meet human expectations [23–26]. Interestingly, although miR-155-5p was low expression and miR-34a-5p was upregulated in senescence endothelial cell in the present research, several researches have reported that miR-155 [27] and miR-34a [28] were downregulation of miRNAs in senescence endothelial cell. Also, the present manuscript was designed to use fibroblasts from mouse skin fibroblasts. Different sources of cells used in their studies might account for the discrepant results with our study. The approach used to establish the senescence endothelial cell model likely affected the results. We used D-galactose (D-gal)-induced cellular senile model because oxidative stress caused by D-galactose is similar to that observed in aging process [29, 30]. The use of the same model to investigate miRNAs expression in the skin could help clarify the roles of these miRNAs.

In this research, we surveyed an obviously increased in the miR-326-3p expression in senescence endothelial cell-derived extracellular vesicles. Extracellular vesicles derived from endothelial cells could not only enhance miR-326-3p expression in skin fibroblasts, but also accelerate skin fibroblasts senescence and apoptosis. Transfection of miR-326-3p mimic into mouse skin fibroblasts significantly promoted cellular senescence and apoptosis. Furthermore, the effect of miR-326-3p mimic was proven to be mediated via targeting Toll-like receptor 4(TLR4). TLR4 was decrease in senescence fibroblasts and aging skin tissue. Our results indicate that extracellular vesicles acts as a carrier of miR-326-3p between endothelial cells and fibroblast cells to coordinate the skin aging process, which demonstrates the cell-to-cell communication between endothelial cell and skin fibroblast cell during aging process. Therefore, targeting endothelial cell-derived extracellular vesicles in antiaging therapies might be a new remedy tactics for delay skin aging.

Furthermore, we only detected the upregulation of miR-326-3p in skin fibroblasts after cocultured with senescence endothelial cell and their extracellular vesicles in this study;, the other miRNAs in the senescence endothelial cell-derived extracellular vesicles have yet to be defined. Hence, they are likely to be secreted into the bloodstream and then passed to skin tissues other than skin fibroblasts, and senescence endothelial cell regulation inevitably leads to an increase in their composition, a result that cannot be disputed.

Evidence showed that cellular senescence is mainly dependent on extracellular vesicles [6, 31–33]. Thus, the function of miR-326-3p-mediated by endothelial cell-extracellular vesicles in the skin aging process is warranted in this study. In this study, miR-326-3p interfered with the TLR4 signaling pathway, resulting in the acceleration of skin fibroblast senescence. TLR4 mRNA expression was weakened by miR-326-3p. There is a strong link between TLR4 and apoptosis in cells [34]. In the last report, miR-21 overexpression inhibits TLR4/NF-*κ*B pathway and reduces apoptosis levels and inflammation incidence in rat cardiomyocytes [35]. Overexpression of miR-200b improved hepatic fibrosis by regulating TLR4 in vivo [36]. As a result, the collection between miR-326-3p and TLR4 expression was not previously described. Therefore, our studies have discovered a novel TLR4 regulatory mechanism.

In conclusion, we demonstrated that senescence endothelial cell-derived extracellular vesicles mediated miR-326-3p to modulate skin fibroblasts function via targeting TLR4. Further studies should investigate endothelial cell-derived extracellular vesicles as a potential novel curative target in skin aging.

## Data Availability

The data used to support the findings of this study are included within the article.
